# HER2-targeted therapy influences CTC status in metastatic breast cancer

**DOI:** 10.1007/s10549-020-05687-2

**Published:** 2020-05-20

**Authors:** Thomas M. Deutsch, Sabine Riethdorf, Carlo Fremd, Manuel Feisst, Juliane Nees, Chiara Fischer, Andreas D. Hartkopf, Klaus Pantel, Andreas Trumpp, Florian Schütz, Andreas Schneeweiss, Markus Wallwiener

**Affiliations:** 1 Department of Gynecology and Obstetrics, University Hospital Heidelberg, Im Neuenheimer Feld 440, 69120 Heidelberg, Germany; 2 Institute of Tumor Biology, University Hospital Hamburg-Eppendorf, Martinistraße 52, 20246 Hamburg, Germany; 3 Department of Medical Oncology, National Center for Tumor Diseases, Im Neuenheimer Feld 460, 69120 Heidelberg, Germany; 4 Institute of Medical Biometry and Informatics, University of Heidelberg, Im Neuenheimer Feld 130.3, 69120 Heidelberg, Germany; 5 Department of Gynecology and Obstetrics, University Hospital Tübingen, Calwerstraße 7, 72076 Tübingen, Germany; 6 Division of Stem Cells and Cancer, German Cancer Research Center (DKFZ), Im Neuenheimer Feld 280, 69120 Heidelberg, Germany; 7 Heidelberg Institute for Stem Cell Technology and Experimental Medicine (HI-STEM gGMBH), Im Neuenheimer Feld 280, 69120 Heidelberg, Germany; 8 German Cancer Research Center (DKFZ), Im Neuenheimer Feld 280, 69120 Heidelberg, Germany

**Keywords:** Metastatic breast cancer (MBC), Human epidermal growth factor receptor 2 (HER2), Circulating tumor cells (CTC), HER2-targeted therapy

## Abstract

**Purpose:**

As an independent, negative-prognostic biomarker for progression-free survival (PFS) and overall survival (OS), circulating tumor cells (CTCs) constitute a promising component for developing a liquid biopsy for patients with metastatic breast cancer (MBC). The effects of HER2-targeted therapy such as trastuzumab, pertuzumab, T-DM1, and lapatinib on CTC status and longitudinal enumeration were assessed in this trial.

**Methods:**

CTC status of 264 patients with MBC was analyzed prior to and after 4 weeks of a new line of palliative systemic therapy. CTCs were assessed using CellSearch®. Three groups were compared: patients with HER2-positive MBC receiving ongoing HER2-targeted therapy (*n* = 28), patients with de novo HER2-positive MBC and no HER2-targeted therapy in the last 12 months prior to enrollment and start of HER2-targeted therapy (*n* = 15), and patients with HER2-nonamplified disease and no HER2-targeted therapy (*n* = 212).

**Results:**

Positive CTC status (≥ 5 CTC/7.5 ml blood) at enrollment was observed in the 3 groups for 17.9, 46.7, and 46.2% (*p* = 0.02) of patients, respectively. At least one CTC/7.5 ml was seen in 28.6, 53.3, and 67.0% (*p* < 0.001) of these patients. Furthermore, 3.6, 40.0, and 3.3% (*p* < 0.001) of the patients had at least one HER2-positive CTC. After 4 weeks of therapy 7.1, 0.0, and 31.1% (*p* = 0.001) of patients had still a positive CTC status (≥ 5 CTC/7.5 ml blood). At least one CTC/7.5 ml was still observed in 25.0, 20.0, and 50.5% (*p* = 0.004) of the patients. Furthermore, 7.1, 0.0, and 1.9% (*p* = 0.187) had at least one HER2-positive CTC. After 3 months of therapy, 35.7, 20.0, and 28.3% (*p* = 0.536) showed disease progression.

**Conclusions:**

HER2-targeted therapy seems to reduce the overall CTC count in patients with MBC. This should be taken into account when CTC status is used as an indicator for aggressive or indolent metastatic tumor disease.

## Background

Worldwide, metastatic breast cancer (MBC) is a major cause of cancer-related death in women [1–3]. MBC, also called stage IV breast cancer, is considered to be not curable [4]. The intent of therapies is therefore palliative and meant to stabilize the disease with tolerable side effects for the patients. The human epidermal growth factor receptor 2 (HER2) gene is overexpressed in about 10–30% of patients with invasive breast cancer [5–7]. In the past, HER2 gene expression was correlated with poor clinical outcome in early breast cancer and metastatic disease alike [8–10]. Since the development of HER2-targeted therapies such as trastuzumab, pertuzumab, T-DM1, and lapatinib, prognosis has changed dramatically. In hormone-receptor positive primary breast cancer, HER2 positivity now even represents a favorable predictor for overall survival (OS) [5]. Newfound HER2 overexpression in biopsies of metastases allow additional, well-tolerated therapy options in the metastasized situation.

Apart from solid metastases, a liquid biopsy as detector and surrogate of the systemic tumor burden is needed to take into account the heterogeneity of the disease [11]. As an independent, negative-prognostic biomarker for progression-free survival (PFS) and OS, circulating tumor cells (CTCs) constitute a promising component for developing a liquid biopsy for patients with MBC [12–14]. Indeed, CTCs are a versatile tool in clinical therapy management and can distinguish between aggressive and indolent metastatic tumor disease [15–17]. CTC monitoring as a prognostic tool in MBC has therefore been introduced in several guidelines such as ASCO and AGO [18, 19]. However, CTCs could not yet show in clinical trials to be predictive for clinical benefit when used to guide decisions on systemic therapy [18]. Independent of clinical and molecular variables, ≥ 5 CTCs per 7.5 ml blood are regarded as the threshold for stratification [20].

As CTCs reflect a subpopulation of the total tumor cell population, characterization and treatment of CTC might be a promising tool for optimizing therapy [21]. Meng et al. demonstrated the presence of HER2-positive CTCs in patients with HER2-negative primary tumors [22]. HER2-positive CTC even proved to be a relevant prognostic factor, independent of primary tumor and metastatic phenotype [23]. However, the therapeutic and predictive relevance of HER2-positive CTC phenotypes is still the subject of controversial discussion [24], and more clinical trials are needed to evaluate their clinical significance.

Retrospective studies and xenograft models suggest that HER2-targeted monoclonal antibody therapies might even be able to target both HER2-nonamplified cancer cells and cancer stem cell populations via antibody-dependent, cell-mediated cytotoxicity (ADCC) [25]. In a phase-II trial, trastuzumab was able to eliminate CTCs independent of HER2 status and decreased the incidence of clinical relapses [26]. On the other hand, another phase-II trial with the intention to treat CTCs in HER2-nonamplified, nonmetastatic breast cancer had to be stopped and showed that trastuzumab does not decrease the detection rate of CTCs [27].

To further investigate these controversial findings, this retrospective study was conducted to demonstrate the immediate effect of HER2-targeted therapies on CTCs in the metastatic setting.

## Patients and methods

**Fig. 1:**
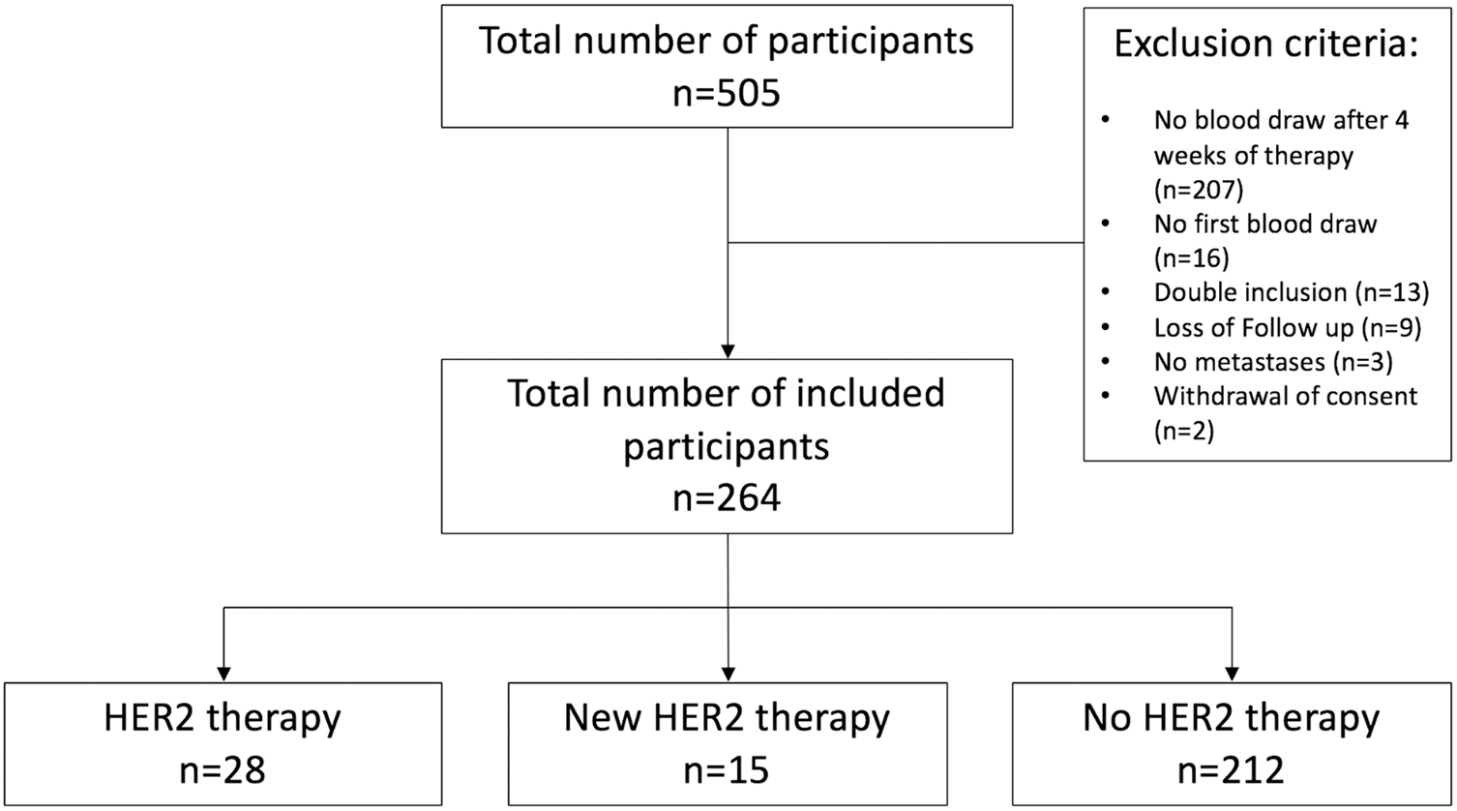
Flow of patients

The CTC study of the National Center for Tumor Diseases (NCT) enrolls patients before administering a new line of systemic therapy when MBC or progressive disease (PD) of MBC is diagnosed. Blood draws are performed at enrollment and 4 weeks after starting a new line of systemic therapy. Since March 2010, 505 patients were enrolled (Fig. 1). In this retrospective trial, all patients for whom CTC data were recorded at baseline and after 4 weeks of therapy were included (*n* = 264). Exclusion criteria were: no blood draw after 4 weeks of therapy (*n* = 207), no blood draw at enrollment (*n* = 16), patients who were already included in the trial (*n* = 13), no follow-up information (*n* = 9), no metastatic disease (*n* = 3), or withdrawal of patient’s consent to participate in the study (*n* = 2). Furthermore, ≥ 5 CTCs per 7.5 ml peripheral blood was defined as CTC-positive [20]. Criteria for inclusion were measurable progressive metastatic disease according to the Response Evaluation Criteria in Solid Tumors (RECIST) criteria [28], age > 18 years, and written informed consent to participate in the study. Ethical approval was obtained from the Ethics Committee of the Medical Faculty of the University of Heidelberg, approval no. S-295/2009.

The study population was divided into 3 groups: patients with HER2-positive MBC with HER2-targeted therapy in the new and previous therapy line (“HER2 therapy”, *n* = 28); patients with HER2-positive MBC and no HER2-targeted therapy in the last 12 months prior to enrollment and start of HER2-targeted therapy (“New HER2 therapy”, *n* = 15); and patients with HER2-nonamplified disease and no HER2-targeted therapy (“No HER2 therapy”, *n* = 212). The “New HER2 therapy” group included patients that were pretreated with chemotherapy and/or endocrine therapy for MBC, since 5–20% of patients show newfound HER2 overexpression in biopsies of metastases [29–31].

Therapy response was evaluated every 3 months via CT and/or MRI scan and categorized according to the Response Evaluation Criteria in Solid Tumors (RECIST) as progressive disease (PD), stable disease (SD), complete remission (CR), or partial response (PR) [28].

Peripheral blood for enumerating CTCs was collected in 7.5 ml CellSave tubes (J Janssen Diagnostics, LLC, Raritan, NJ, USA). The blood samples were processed and analyzed within 96 h, using the CellSearch™ assay (CellSearch™ Epithelial Cell Kit/CellSpotter™ Analyzer, Janssen Diagnostics, LLC, Raritan, NJ, USA), strictly following the manufacturer’s instructions. The CellSearch™ assay uses epithelial cell adhesion molecule (EpCAM)-based immunomagnetic enrichment and immunofluorescence with antibodies against keratins and CD45, differentiating between debris, hematopoietic cells, and epithelial cells [32], and provides high intra-observer, interobserver, and inter-instrument agreement that led to FDA approval [33–36]. EpCAM-positive cells were labeled with the nuclear dye 4′,6-diamidino-2-phenylindole (DAPI) and immunostained with monoclonal keratin and CD45-specific antibodies. The CellSpotter™ Analyzer was used by trained staff to detect CTCs as previously described [37–40]. Here, ≥ 5 CTCs per 7.5 ml blood was considered as CTC-positive [20]. The anti-HER2 antibody fluorescein isothiocyanate (FITC, CellSearch tumor-phenotyping reagent HER2, Janssen Diagnostics LLC, Raritan, NJ, USA) was used to characterize HER2 expression in CTCs by applying the CellSearch technology, as described previously [19, 26, 27]. HER2 score was determined according to the intensity of HER2-specific immunofluorescence and characterized as negative (0), weak (1+), moderate (2+), or strong (3+). CTC status was considered HER2-positive if at least one CTC exhibited strong (3+) or moderate (2+) HER2 staining [8].

Clinical characteristics of the cohort were described as absolute and relative frequencies for binary and ordinal variables, as well as mean and 95% confidence intervals for continuous variables. Mean PFS and OS times were estimated counting from the timepoint of study enrollment. Differences between groups were analyzed by Chi-squared tests (categorical data) and *t* tests (continuous variables). Statistical analyses were performed using R (version 3.6.0) [41]. Figures were generated using Microsoft Office Version 16.30. Since this is an exploratory study, *p* values should be interpreted in a descriptive sense. *p* values smaller than 0.05 were defined as significant.

## Results

**Fig. 2:**
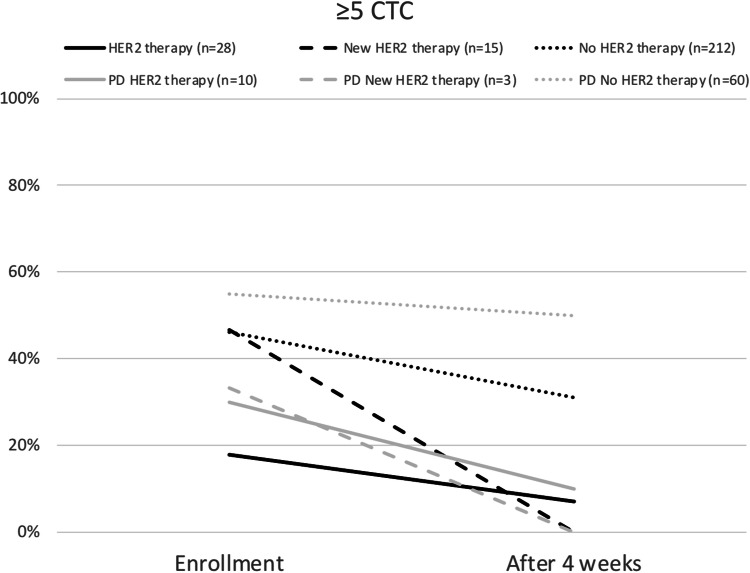
Rate of patients with ≥ 5 CTCs at enrollment and after 4 weeks of therapy and patients with progression of disease (PD) after 3 months

**Fig. 3:**
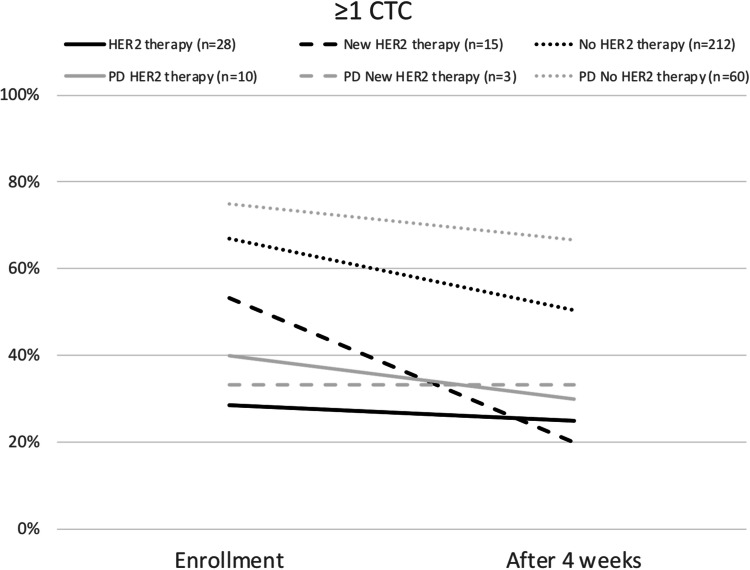
Rate of patients with ≥ 1 CTC at enrollment and after 4 weeks of therapy and patients with progression of disease (PD) after 3 months

Initially, CTC-positive (≥ 5 CTC/7.5 ml blood) were 17.9, 46.7, and 46.2% (*p* = 0.02) of patients in the three groups “HER2 therapy”, “New HER2 therapy”, and “No HER2 therapy” as shown in Table 1. At least one CTC/7.5 ml was detected in 28.6, 53.3, and 67.0% (*p* < 0.001) of these patients. In total 3.6, 40.0, and 3.3% (*p* < 0.001) of the study population had at least one CTC with HER2 positivity. After 4 weeks of therapy, 7.1, 0.0, and 31.6% (*p* = 0.001) of patients were still CTC-positive. The black bars in Fig. 2 demonstrate the trend of CTC positivity under therapy. At least one CTC/7.5 ml was detected in 25.0, 20.0, and 50.5% (*p* = 0.004) of the patients in the three groups after 4 weeks of therapy. This trend is visualized in Fig. 3. At this timepoint 7.1, 0.0, and 1.9% (*p* = 0.187) of the patients had at least one CTC showing HER2 expression.

**Table 1:** Patient characteristics and rate of CTC status divided by therapy groups after enrollment

		HER2 therapy	New HER2 therapy	No HER2 therapy	*p*
Total	*n*	28	15	212	
≥ 1 CTC at enrollment	Rate	28.6%	53.3%	67.0%	< 0.001
≥ 5 CTC at enrollment	Rate	17.9%	46.7%	46.2%	0.02
≥ 1 HER2-positive CTC at enrollment	Rate	3.6%	40.0%	3.3%	< 0.001
≥ 1 CTC after 4 weeks	Rate	25.0%	20.0%	50.5%	0.004
≥ 5 CTC after 4 weeks	Rate	7.1%	0.0%	31.6%	0.001
≥ 1 HER2-positive CTC after 4 weeks	Rate	7.1%	0.0%	1.9%	0.187
PD after 3 months	*n*	10	3	60	
	Rate	35.7%	20.0%	28.3%	0.536
PD + ≥ 1 CTC at enrollment	Rate	40.0%	33.3%	75.0%	0.036
PD + ≥ 5 CTC at enrollment	Rate	30.0%	33.3%	55.0%	0.284
PD + ≥ 1 HER2 positive CTC at enrollment	Rate	10.0%	33.3%	5.0%	0.151
PD + ≥ 1 CTC after 4 weeks	Rate	30.0%	33.3%	66.7%	0.056
PD + ≥ 5 CTC after 4 weeks	Rate	10.0%	0.0%	50.0%	0.019
PD + ≥ 1 HER2-positive CTC after 4 weeks	Rate	0.0%	0.0%	5.0%	0.713
Age at diagnosis BC	Mean (95% CI)	46.6 years (42.2–51.0)	54.9 years (50.6–59.2)	52.2 years (50.7–53.8)	0.032
Age at enrollment	Mean (95% CI)	54.7 years (50.2–59.1)	59.1 years (54.5–63.7)	59.3 years (59.1–59.5)	0.059
Number of previous lines of CHT for MBC	Mean (95% CI)	1.9 (1.3–2.5)	0.4 (0.2–0.7)	1.5 (1.3–1.7)	0.570
Previous endocrine therapy for MBC	Rate	39.3%	20.0%	52.4%	0.033
PFS	Mean (95% CI)	8.8 months (5.7–11.8)	14.5 months (5.4–23.7)	10.6 months (8.7–12.4)	0.755
OS	Mean (95% CI)	26.1 months (19.8–32.3)	42.7 months (33.0–52.5)	26.8 months (23.9–29.6)	0.526

Progression of disease (PD) after 3 months of therapy in the study was observed for 10 (35.7%), 3 (20.0%), and 60 (28.3%) patients, respectively, in the three treatment groups (*p* = 0.536). These patients showed higher rates of CTCs after 4 weeks of therapy than patients with at least stable disease. In all, 10.0, 0.0, and 50.0% (*p* = 0.019) of these patients were CTC-positive after 4 weeks of therapy if disease had progressed. At least one CTC/7.5 ml was detected in 30.0, 33.3, and 66.7% (*p* = 0.056) among those patients with PD. These trends are depicted with the gray bars in Figs. 2 and 3.

Regarding PFS and OS, patients with “New HER2 therapy” had the best prognosis, with a mean PFS of 14.5 months (95% confidence interval [CI] 5.4–23.7) and mean overall survival of 42.7 months (95% CI 33.0–52.5) for OS followed by “No HER2 therapy” with 10.6 months (95% CI 8.7–12.4) and 26.8 months (95% CI 23.9–29.6), respectively, and patients under ongoing “HER2 therapy” with 8.8 months (95% CI 5.7–11.8) and 26.1 months (95% CI 19.8–32.3). The “New HER2 therapy” group had received the fewest lines of chemotherapy previously (mean 0.4, 95% CI 0.2–0.7) followed by “No HER2 therapy” (mean 1.5, 95% CI 1.3–1.7) and “HER2 therapy” (mean 1.9, 95% CI 1.3–2.5).

In all, 144 (67.9%) patients of the “No HER2 therapy” group received chemotherapy with palliative intention before being enrolled in the study, 19 (9.0%) patients with metastatic disease had received only endocrine therapy, and 49 (23.1%) patients had not received any palliative therapy before enrollment (Table 2). Furthermore, 54.2% of the chemotherapy group had a positive CTC status at enrollment, 73.6% ≥ 1 CTC and 2.8% ≥ 1 HER2-positive CTC, compared to 26.3% positive CTC status, 52.6% ≥ 1 CTC and 0.0% ≥ 1 HER2-positive CTC of the endocrine therapy group, and 30.6% positive CTC status, 53.1% ≥ 1 CTC and 6.1% ≥ 1 HER2-positive CTC in the group with no previous treatment. After 4 weeks of therapy (chemotherapy or endocrine therapy), 36.8% of the group that had received chemotherapy before enrollment had a positive CTC status, 57.6% ≥ 1 CTC and 2.8% ≥ 1 HER2-positive CTC, compared to 10.5% positive CTC status, 26.3% ≥ 1 CTC and 0.0% ≥ 1 HER2-positive CTC of the group that received endocrine therapy before enrollment, and 22.4% positive CTC status, 38.8% ≥ 1 CTC and 0.0% ≥ 1 HER2-positive CTC with no previous treatment before enrollment. In these 3 groups, 32.6, 15.8, and 20.4%, respectively, experienced PD after 3 months. PFS was 10.4 months (95% CI 8.0–12.8), 13.4 months (95% CI 7.3–19.5), and 9.9 months (95% CI 7.0–12.8), respectively, in the three groups. OS was 24.8 months (95% CI 21.1–28.5), 30.5 months (95% CI 22.3–38.8), and 31.0 months (95% CI 26.0–35.9), respectively.

**Table 2:** Patient characteristics and rate of CTC status of HER2-nonamplified patients (No HER2 therapy) divided by palliative therapy before enrollment

		Chemotherapy before enrollment	Endocrine therapy before enrollment	No therapy before enrollment	*p*
Total	*n*	144	19	49	
≥ 1 CTC at enrollment	Rate	73.6%	52.6%	53.1%	0.012
≥ 5 CTC at enrollment	Rate	54.2%	26.3%	30.6%	0.003
≥ 1 HER2-positive CTC at enrollment	Rate	2.8%	0.0%	6.1%	0.369
≥ 1 CTC after 4 weeks	Rate	57.6%	26.3%	38.8%	0.006
≥ 5 CTC after 4 weeks	Rate	36.8%	10.5%	24.5%	0.032
≥ 1 HER2-positive CTC after 4 weeks	Rate	2.8%	0.0%	0.0%	0.062
PD after 3 months	*n*	47	3	10	
	Rate	32.6%	15.8%	20.4%	0.116
Age at diagnosis BC	Mean (95% CI)	50.0 years (48.3–51.7)	56.5 years (51.5–61.4)	55.0 years (51.6–58.5)	0.014
Age at enrollment	Mean (95% CI)	58.7 years (56.9–60.6)	63.3 years (58.2–68.4)	59.5 years (56.1–62.8)	0.513
Number CHT met	Mean (95% CI)	2.2 (1.9–2.4)	0 (0.0–0.0)	0 (0.0–0.0)	< 0.001
Endocrine therapy met	Rate	63.2%	100%	0%	< 0.001
PFS	Mean (95% CI)	10.4 months (8.0–12.8)	13.4 months (7.3–19.5)	9.9 months (7.0–12.8)	0.955
OS	Mean (95% CI)	24.8 months (21.1–28.5)	30.5 months (22.3–38.8)	31.0 months (26.0–35.9)	0.065

Table 3 demonstrates the different anti-HER2 therapies that were administered after enrollment in the study. Of the patients under ongoing HER2 therapy, 12 patients received trastuzumab + chemotherapy (eribulin, vinorelbin, paclitaxel, or nab-paclitaxel), 3 patients trastuzumab + pertuzumab + chemotherapy (docetaxel or paclitaxel), 3 patients trastuzumab + lapatinib, T-DM1, and 6 patients received lapatinib + chemotherapy (capecitabine or MTX + cyclophosphamide). After 3 months of therapy, 3 patients who had received trastuzumab + chemotherapy (eribulin or vinorelbin), all 4 patients with T-DM1, and 3 patients with lapatinib + chemotherapy (capecitabine or MTX + cyclophosphamide) showed PD. A decrease in CTCs was shown for trastuzumab + chemotherapy (eribulin, vinorelbin, or paclitaxel) (*n* = 5), trastuzumab + pertuzumab + paclitaxel (*n* = 1), and lapatinib + capecitabine (*n* = 1). An increase in CTCs was observed for trastuzumab + vinorelbin (*n* = 1), trastuzumab + lapatinib (*n* = 1), and lapatinib + capecitabine (*n* = 2). In the “New HER2 therapy” group, 3 patients received trastuzumab + chemotherapy (docetaxel or vinorelbin), 5 patients trastuzumab + pertuzumab + chemotherapy (docetaxel, paclitaxel, or vinorelbin), 1 patient trastuzumab + pertuzumab, 4 patients T-DM1, and 2 patients pertuzumab + paclitaxel. PD was observed in 2 patients who had received T-DM1 and in 1 patient with pertuzumab + paclitaxel. A decrease in CTCs was shown for trastuzumab + docetaxel (*n* = 1), trastuzumab + pertuzumab + docetaxel (*n* = 3), trastuzumab + pertuzumab (*n* = 1), and T-DM1 (*n* = 3). No increase in CTCs was observed in the “New HER2 therapy” group.

**Table 3:** Number of patients with HER2-targeted therapies and combined chemotherapies after enrollment. Number of patients with progression of disease (PD) under anti-HER2 therapy

Anti-HER2 therapy	Chemotherapy		HER2 therapy *n*	New HER2 therapy *n*
Trastuzumab	Docetaxel/Eribulin/Vinorelbin/ Paclitaxel/nab-Paclitaxel	Total	12	3
		PD	3	0
Trastuzumab + Pertuzumab	Docetaxel/Paclitaxel/ Vinorelbin	Total	3	5
		PD	0	0
Trastuzumab + Pertuzumab	No	Total	0	1
		PD	0	0
Trastuzumab + Lapatinib	No	Total	3	0
		PD	0	0
T-DM1	No	Total	4	4
		PD	4	2
Lapatinib	Capecitabin/ MTX + Cyclophosphamid	Total	6	0
		PD	3	0
Pertuzumab	Paclitaxel	Total	0	2
		PD	0	1

## Discussion

In this analysis, the CTC status of patients with MBC, stratified by ongoing therapy and previous treatment, was investigated. The point prevalence of CTC at enrollment was highest in patients receiving ongoing palliative chemotherapy for HER2-nonamplified stage IV breast cancer. Throughout all therapy groups, a decrease in CTCs was observed after 4 weeks of a new line of palliative therapy (Figs. 2 and 3). Even for the patients in whom disease had progressed after 3 months of therapy, a tendency for CTCs to decrease is evident, as demonstrated by the gray bars in Figs. 2 and 3. As described in multiple studies previously, less reduction or persistence of CTCs under therapy correlates with PD [13, 16, 39, 42, 43]. Therefore, patients were not only divided in groups with < or ≥ 5 CTCs but also in < or ≥ 1 CTC at enrollment and after 4 weeks of therapy. Looking at the HER2-nonamplified patients, divided by palliative therapy just before enrollment, the subgroups with the highest rates of PD had also higher rates of CTCs at the time of enrollment (Table 2). This supports the theory proposed by Cristofanilli et al., whereby MBC can be divided into stage IV aggressive and stage IV indolent disease depending on CTC status [15]. Nevertheless, patients with HER2-positive disease do not seem to fit to this theory. At enrollment, the “New HER2 therapy” group had significantly higher CTC values than the “HER2 therapy” group (≥ 1 CTC; 53.3 vs. 28.6% and ≥ 5 CTCs; 46.7 vs. 17.9%; *p* < 0.001, respectively, *p* = 0.02). In contrast, the PFS and OS were significantly better here with 14.5 vs. 8.8 months and 42.7 vs. 26.1 months. This was accompanied by a decrease in ≥ 1 CTC rate of 23.3% (vs. 3.6%) and ≥ 5 CTCs rate of 46.7% (vs. 10.8%) within 4 weeks of therapy (Figs. 2 and 3). Of note is also the significantly higher rate of HER2-positive CTCs in the “New HER2 therapy” group at enrollment (40.0 vs. 3.6% [*p* < 0.001]) and the reduction to 0% (vs. 7.1% [*p* = 0.187]) after 4 weeks of HER2-targeted therapy (Table 1). It needs to be considered that the “HER2 therapy” group had a mean of 1.9 (range 1.3–2.5) previous palliative chemotherapy lines, whereas the “New HER2 therapy” group had a mean of only 0.4 (range 0.2–0.7) lines of previous chemotherapy in the metastatic situation. This partly explains the advantages in PFS and OS. These findings are conclusive with Giardano et al., who stated that the baseline CTC count of HER2-positive patients is least valuable for prognostic prediction suggesting an interaction between CTCs and HER2-targeted therapies [44]. However, they didn’t distinguish between ongoing palliative HER2-targeted treatment and de novo HER2-positive MBC. Regarding only patients with first-line chemotherapy for MBC, Pierga et al. revealed that CTC decrease seems strongest under targeted therapy [45]. These findings were also confirmed by our results.

The impact of CTC status in de novo MBC and the correlation between first-line HER2-targeted therapy vs. first-line endocrine and chemotherapy is apparent when comparing the “New HER2 therapy” group with the “No therapy before enrollment” group of the HER2-nonamplified subgroup; the prevalence of ≥ 1 CTC at enrollment was similar (53.3 vs. 53.1%) but the rate of positive CTC status (≥ 5 CTC) at enrollment was higher in the “New HER2 therapy” group (46.7 vs. 30.6%). After 4 weeks of therapy, ≥ 1 CTC were found in 20.0 vs. 38.8% of patients and a positive CTC status was observed in 0 vs. 22.4% of patients. PD rate was similar at 20.0 vs. 20.4%. PFS and OS were better in the “New HER2 therapy” group (14.5 vs. 9.9 months, 42.7 vs. 31.0 months, respectively).

As other retrospective studies and xenograft models have shown that HER2-targeted monoclonal antibody therapies might even be able to target HER2-nonamplified cancer cells and cancer stem cell populations including CTCs, the indication for HER2-targeted therapies might need to be extended [25, 26]. Antibody-dependent, cell-mediated cytotoxicity (ADCC) might impact a subgroup of patients showing heterogenic tumor spread, represented by CTCs. Nevertheless, these findings also demonstrate that low rates of CTC levels do not necessarily correlate with better prognosis in cross-therapy comparisons. Negative phase-II trials, such as the TREAT-CTC trial, with the intention to treat CTCs of HER2-nonamplified patients, illustrate the complexity of the matter [27]. The clinical significance of the CTC phenotype for guiding therapeutic decisions is currently being investigated in the DETECT studies [46].

The clinical relevance of CTC status on prognosis, including HER2-positive patients, is undisputed [15]. These findings lead us to assume that the CTC status might be strongly affected by HER2-targeted therapies but does not necessarily correlate with prognosis in patients with HER2-positive de novo MBC as compared to HER2-nonamplified de novo MBC. Furthermore, the CTC status of HER2-positive patients needs to be evaluated according to the therapy situation (before vs. ongoing anti-HER2 therapy). This is underlined by the observation that patients receiving HER2-targeted therapy have, independent of the prognosis, significantly lower levels of CTCs than patients receiving chemotherapy, endocrine therapy, or no therapy (Fig. 2 and 3). This overall decrease in CTC count must be considered when comparing different therapy groups.

## Limitations

One main limitation lies in the fact that this is a retrospective analysis. Another limitation is the small number of analyzed patients in the “HER2 Therapy” and “New HER2 therapy” groups. Indeed, the small number of patients with PD highlights the implicit difficulties in analyzing the rate of CTC-positive cases. The subgroup receiving anti-HER2 therapy can therefore only be reported descriptively and used to generate new hypotheses about therapy effects on CTC levels.

## Conclusions

First-line HER2-targeted therapy of metastatic breast cancer seems to reduce CTC levels greater than endocrine or chemotherapy. Ongoing anti-HER2 therapy seems to be associated with lower overall CTC levels.
